# Synergistic Effects of Black Phosphorus/Boron Nitride Nanosheets on Enhancing the Flame-Retardant Properties of Waterborne Polyurethane and Its Flame-Retardant Mechanism

**DOI:** 10.3390/polym12071487

**Published:** 2020-07-03

**Authors:** Sihao Yin, Xinlin Ren, Peichao Lian, Yuanzhi Zhu, Yi Mei

**Affiliations:** 1 Faculty of Chemical Engineering, Kunming University of Science and Technology, Kunming 650500, China; yinsihao1213@126.com (S.Y.); lianpeichao@126.com (P.L.); yuanzhi_zhu@kust.edu.cn (Y.Z.); 2 The Higher Educational Key Laboratory for Phosphorus Chemical Engineering of Yunnan Province, Kunming University of Science and Technology, Kunming 650500, China; 3 Yunnan Provincial Key Laboratory of Energy Saving in Phosphorus Chemical Engineering and New Phosphorus Materials, Kunming 650500, China

**Keywords:** black phosphorene, boron nitride, flame retardant, waterborne polyurethane

## Abstract

We applied black phosphorene (BP) and hexagonal boron nitride (BN) nanosheets as flame retardants to waterborne polyurethane to fabricate a novel black phosphorus/boron nitride/waterborne polyurethane composite material. The results demonstrated that the limiting oxygen index of the flame-retarded waterborne polyurethane composite increased from 21.7% for pure waterborne polyurethane to 33.8%. The peak heat release rate and total heat release of the waterborne polyurethane composite were significantly reduced by 50.94% and 23.92%, respectively, at a flame-retardant content of only 0.4 wt%. The superior refractory performances of waterborne polyurethane composite are attributed to the synergistic effect of BP and BN in the gas phase and condensed phase. This study shows that black phosphorus-based nanocomposites have great potential to improve the fire resistance of polymers.

## 1. Introduction

Polymeric materials have been widely used in electronic devices, construction, and transportation. However, most of the polymeric materials have been intrinsically inflammable [1,2]. Therefore, flame-retardant additives are important to mitigate the risk of fire [3,4]. Some halogen-based flame retardants have been banned because they form carcinogens during combustion [5,6,7]. Compared to traditional halogenated flame retardants, phosphorus-containing flame retardants have attracted much attention due to the advantages of having low smoke and low halogen content as well as being non-toxic [8,9,10]. Phosphorus flame retardants can be divided into inorganic phosphorus flame retardants and organophosphorus flame retardants. Organophosphorus flame retardants have low cost and good compatibility with polymers, but they have high volatility and poor thermal stability [11]. Inorganic phosphorus flame retardants have high phosphorus content, high flame retardant efficiency and low toxicity, but their particles are usually large, thus resulting in poor compatibility with polymer materials and uneven dispersion [12]. For example, red phosphorus needs to be modified or coated to increase its compatibility with polymer materials [13,14].

Black phosphorus (BP) is a new kind of 2D layered material that is only composed of the phosphorus element [15,16,17]. Recently, BP has been demonstrated to be a good flame retardant [18,19,20]. The high specific surface area of the layered structure can result in an efficient barrier effect in the process of polymer combustion. Compared with volatile white phosphorus and amorphous red phosphorus, BP also exhibits higher thermal stability and phase-dispersion, which could improve the flame-retardant performance and reduce damage to the mechanical properties of the polymers. In previous research, we reported that BP can effectively enhance the thermal stability and fire resistance of polymers [21]. Yuan Hu, et al. synthesized the BP/carbon nanotube composite and demonstrated its synergistic flame retardant performance for epoxy resin [22]. However, some key indicators of flame-retardant property, such as the limit oxygen index (LOI), for the reported BP-based composite materials need to be further improved.

Hexagonal boron nitride (BN) nanosheet is a widely studied 2D material with high thermal stability, good mechanical strength, and large surface area. It has been found to be a good flame retardant for several polymers [23,24,25]. Considering the fact that the flame-retardant mechanism of BP materials has been mainly attributed to the formed radicals in the gas phase while the BN nanosheet mainly works in the condensed phase, the combination of BP and BN may have a good synergistic effect to further improve the flame-retardant performance and decrease the additive amount of the flame retardants.

**Figure 1:**
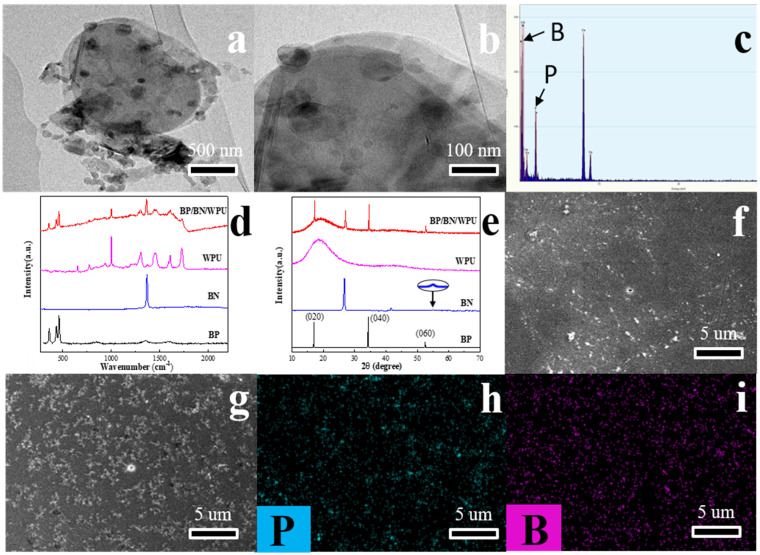
(**a**,**b**) TEM image of BP/BN nanosheets; (**c**) The EDS of BP/BN nanosheets; (**d**) Raman spectra of BP, BN, WPU and BP/BN/WPU; (**e**) XRD spectra of BP, BN, WPU and BP/BN/WPU; (**f**) and (**g**) SEM image of WPU and BP/BN/WPU; (**h**) phosphorus mapping in BP/BN/WPU and (**i**) boron mapping in BP/BN/WPU.

Thus, in this paper, we designed a series of experiments to analyze the synergistic effect and flame-retardant mechanism of BP and BN by filling them into waterborne polyurethane (WPU). Through systematic characterization, we found that adding only 0.4 wt% BP/BN nanosheet could significantly raise the LOI of WPU from 21.7% to 33.8%. The improvement of flame retardancy involves the combination of the condensed phase and gas phase effects during combustion. In addition, such a small additive amount of BP/BN showed negligible color effect on the WPU (Figure 1), which broadens its real application range.

## 2. Materials and Methods

### 2.1. Materials

In this study, BP was prepared by a mineralization transformation method from red phosphorus with the help of iodine and tin in a quartz tube under argon atmosphere. The prepared BP crystals were washed with toluene to remove residual mineralizer, followed by washing with water and acetone. The utilized red phosphorus, iodine, tin, toluene and acetone were analytically pure. The BN was purchased from Shanghai Huayi Group Huayuan Fine Chemicals Co., Ltd., Shanghai, China with a particle size lower than 30 µm. The WPU latex with a solid content of 25 wt% was purchased from Anhui Huatai New Material co. Ltd., Hefei, China The water used was deionized water.

### 2.2. Fabrication of BP and BN Nanosheets

BP was ground for 2 h into powder, and then 0.5 g of the powder was added into a conical flask with 500 mL of deionized water. The conical flask was sealed with argon gas to stop oxidation of the BP. Then the dispersion was added to a working ultrasonic device (50 Hz, 200 W) for 24 h with the temperature controlled below 30 °C. Afterwards, the dispersed liquid was centrifuged at 3500 rpm for 15 min by a centrifugal machine (TGL-16C, Shanghai Anting Scientific Instrument Factory, Shanghai, China). Finally, the supernatant liquid was collected and condensed by suction filtration. An argon atmosphere should be used to prevent the oxidation of phosphorene throughout the whole experimental process. The dispersion of boron nitride was also obtained by liquid phase stripping. In order to calculate the concentration of the dispersion, freeze drying was used to remove the moisture. Then the remaining solid was weighed.

### 2.3. Preparation of BP/BN/WPU Composite Materials

The obtained BP and h-BN dispersion were added into a beaker with WPU. After stirring for a few minutes, the beaker was sealed by filling with an argon atmosphere. The mixture was ultrasonicated for 2 h at low temperature with an ice bath. Then the obtained suspension was poured onto a plate of the size of 120 × 120 mm and dried under vacuum at 22 °C. After it was dried completely, the BP/BN/WPU material was formed. The additive amount of BP/BN is 0.2%/0.2%. For comparison, the BP/WPU with 0.4% BP and BN/WPU with 0.4% BN were synthesized using the same method (Table 1).

### 2.4. Analytical Test

#### 2.4.1. Structure Characterizations

Transmission electron microscopy (TEM, Philips CM100, Amsterdam, The Netherlands) was conducted to observe the BP and BN nanosheets at an acceleration voltage of 100 kV.

An X-ray diffraction device (XRD, PANalytical Empyrean, Almelo, The Netherlands) was used to analyze the BP, BN powders, as well as the WPU and its composite materials, respectively.

Raman spectra were obtained on a LabRAM-HR Confocal Raman Microprobe (HORIBA Scientific Co., Palaiseau, France) with excitation provided in backscattering geometry by a 633 nm argon laser line.

Scanning electron microscopy (SEM, Bruker Nano, Bruker, Karlsruhe, Germany) was used to analyze the microstructure of the materials and the charred residue. The WPU and its composite materials with BP and BN nanosheets were fractured by liquid nitrogen, then the fracture surfaces of the samples were uniformly coated with a layer of gold and then observed by SEM. In order to present the distribution of phosphorene in the polymers, elemental mapping tests were also conducted on another two specimens without a coating of a gold layer. In addition, An EDS test of the charred residue was applied to determine the content of BP and BN in the condensed phase.

X-ray photoelectron spectroscopy (XPS) analysis was performed using Al radiation as a probe (K-alpha, Thermo Fisher Scientific, Waltham, MA, USA) to measure the valence states and chemical composition of the residue of BP/BN/WPU.

#### 2.4.2. Thermal Properties Measurement

Thermogravimetric analysis (TG) of the materials was performed on a thermal analyzer (NETZSCH STA449F3, NETZSCH, Selb, Germany) with a gas flow rate of 80 mL/min under nitrogen atmosphere. The heating rate was 10 °C/min and the temperature ranged from 40 °C to 800 °C.

Thermogravimetric analysis–Fourier transform infrared spectroscopy (TG–FTIR) was performed via a TGA/DSC3 thermogravimetric analyzer (METTLER TOLEDO, Greifensee, Switzerland) linked with a Nicolet FTIR IS50 spectrometer (Thermo Fisher) at a heating rate of 10 °C/min within a temperature range of 40 °C to 800 °C under a N_2_ flow of 50 mL/min. The temperature of the transfer line in the TG–FTIR system was 200 °C.

#### 2.4.3. Flammability Property Measurement

The limiting oxygen index (LOI) values were measured according to the standard oxygen index test ASTM D2863-77 by using the device of COI from Motis combustion technology co. LTD (Kunshan, China).

In order to study the combustion behavior, cone calorimetry (CC, PX-07-007, Suzhou phoenix quality inspection instrument co. LTD, Suzhou, China) was performed at a heat flux of 35 kW/m^2^. The material specimens were made into a square with the size of 100 × 100 × 3 mm. After wrapping in a piece of aluminum film, the specimens were set on fire on the CC.

## 3. Results and Discussion

### 3.1. Characterization of BP/BN/WPU

The TEM images reveal the micromorphology of BP and BN nanosheets prepared by liquid phase stripping. As shown in Figure 1a,b, it can be clearly seen that the bulk black phosphorus and BN were peeled into a few layers of nanoflakes. We suggest that the BP nanosheets are arranged on the BN nanosheets surface, which were fully stripped with clear edges with a length of several micrometers. The Energy Dispersive Spectrometer (EDS) results (Figure 1c) confirmed the elements’ distribution in the BP/BN nanosheets. Raman spectra and X-ray diffraction (XRD) were carried out for BP, BN powder, pure WPU and BP/BN/WPU composite, respectively. As shown in the Raman spectra (Figure 1d), the BP nanosheets show three characteristic peaks of A_g_^1^, B_g_^2^, and A_g_^2^, corresponding to the crystal orientation [26], thickness [27], and angle [28]. The BN shows the in-plane ring vibration peak of BN (E2g vibration mode) at 1365 cm^−1^ [29]. The BP/BN/WPU composite shows four new peaks in comparison with pure WPU, indicating the successful introduction of BP and BN nanosheets into the WPU matrix. The XRD spectrum of BP reveals three obvious diffraction peaks (Figure 1e), corresponding to the (020), (040), and (060) plane, respectively [30,31]. The spectrum of BN also has three obvious diffraction peaks, indicating the good crystallinity of BN. The XRD spectrum of BP/BN/WPU retains the main peaks of BP and BN, which is consistent with the Raman results.

The SEM images of pure WPU (Figure 1f) are observed on the fractured surface. It is shown to be homogeneous and without any additive particles due to the typical fracture behavior of a homogeneous material. Compared with the pure WPU, BP/BN/WPU composite shows several white nanosheets (Figure 1g), which could be attributed to the additives BP and BN. As shown in Figure 1h–i, both the P and B elements distribute uniformly over a large region for the BP/BN/WPU sample. These results indicated that the BP and BN nanosheets were uniformly distributed into the WPU.

### 3.2. Thermal Stability of BP/BN/WPU and Its Nanocomposite

**Figure 2:**
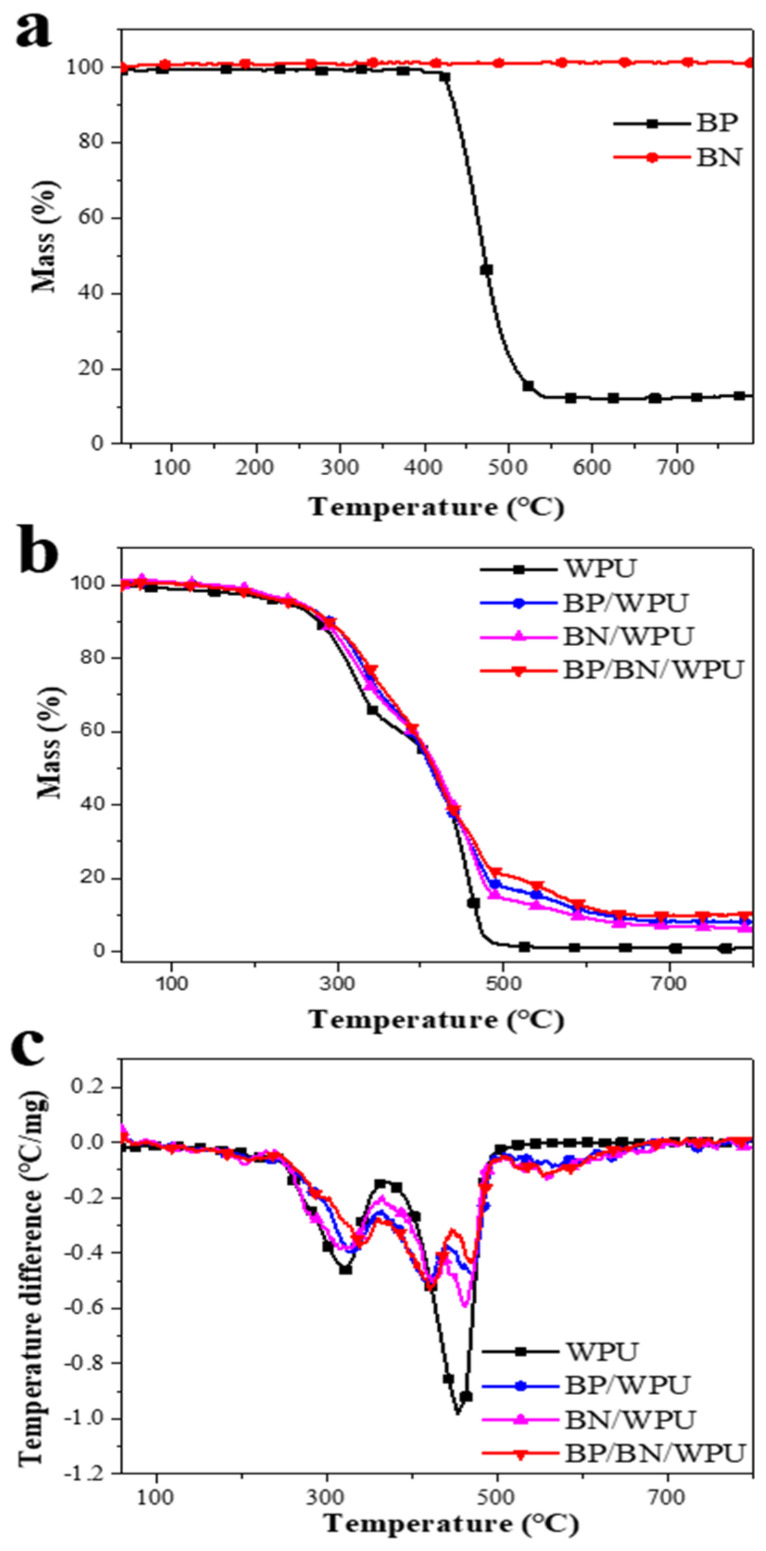
(**a**) the TGA curves of BP and BN; (**b**) the TGA curves of WPU, BP/WPU, BN/WPU and BP/BN/WPU; and (**c**) the resulted DTG curves of WPU, BP/WPU, BN/WPU and BP/BN/WPU.

The TG analysis of the specimens was carried out in a N_2_ atmosphere to analyze the decomposition behavior in a real fire scenario. As shown in Figure 2a, the TG curves of pure BP have an obvious mass loss starting at 420 °C, while the BN only shows negligible mass loss over the whole temperature range. These results show that the BP turns into the gas phase after 420 °C, and the BN is very stable even at higher temperature. From Figure 2b,c, we can see that the WPU shows two major mass loss stages over the selected temperature range. This is caused by the difference in thermal stability between the hard segment and soft segment of WPU. The first mass loss stage is mainly caused by the breakage of urethane bonds in the amine and isocyanate while the second stage is assigned to the decomposition of residual polyols. In contrast to pure WPU, the BP/WPU, BN/WPU and BP/BN/WPU exhibited three main mass loss stages. The third stage of the composite occurred in the range of 440–500 °C, which indicates that the mass loss became slower at this temperature after the addition of BP and BN nanosheets. The residue char of BP/BN/WPU reached 10.11%, which is also higher than that of WPU (0.93%), BP/WPU (8.11%), and BN/WPU (6.44%). These results revealed that the BP and BN nanosheets can synergistically improve the thermal stability of WPU and promote the formation of residue char [32].

### 3.3. Fire Safety Properties of BP/BN/WPU

**Figure 3:**
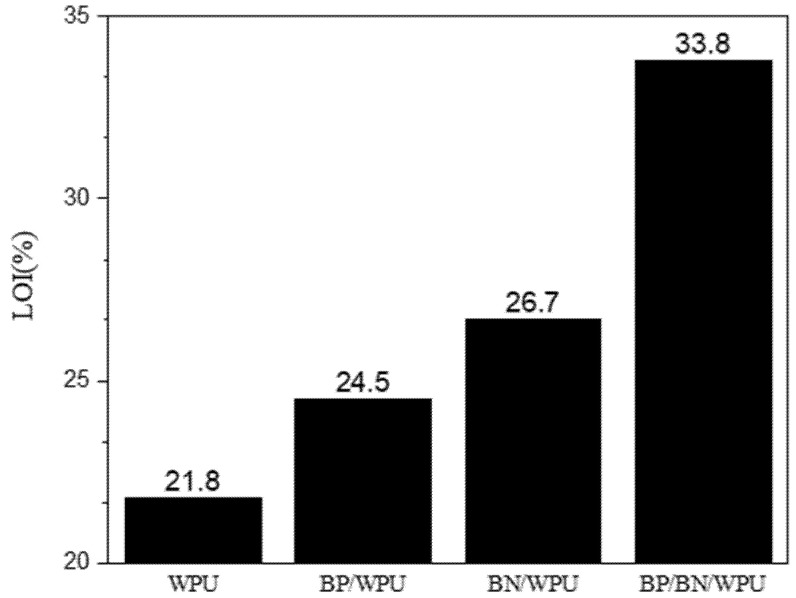
The additive amount of the flame retardant and values of the limit oxygen index (LOI) of the samples.

The limiting oxygen index (LOI) test is used to determine the flammability of the samples. As depicted in Figure 3, the LOI of pure WPU is 21.8%, indicating that the polymeric matrix is a flammable material. Adding 0.4 wt% of BP or BN increased the LOI to 24.5% for 0.4%BP/WPU and 26.7% for 0.4%BN/WPU, respectively. Interestingly, simultaneous addition of 0.2% of BP and BN into WPU significantly increase the LOI to 33.8% and reached the V0 of fire resistance. That is to say, BP and BN have a synergistic effect in enhancing the self-extinguishing ability of WPU in a real fire scenario.

**Figure 4:**
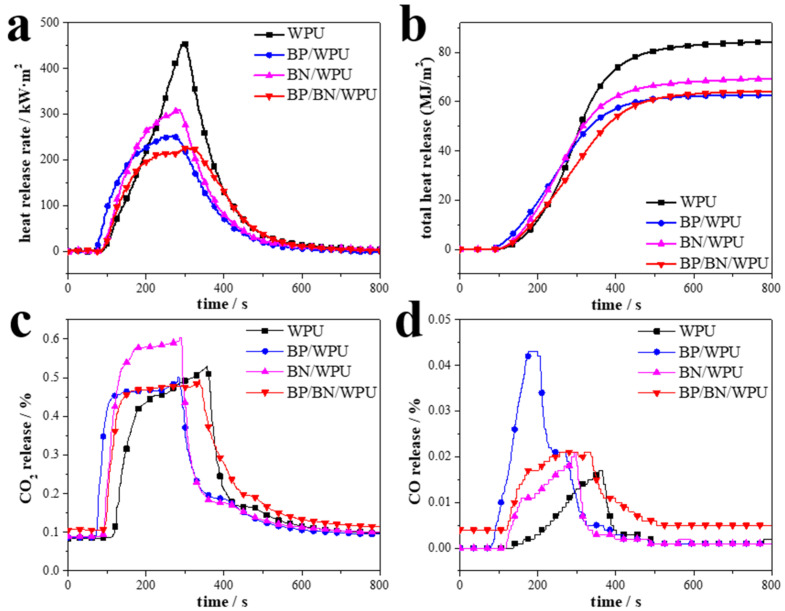
Cone calorimeter test of the samples: (**a**) HRR curves; (**b**) THR curves; (**c**) CO_2_ release curves; (**d**) CO release curves.

The cone calorimeter (CC) tests were applied to simulate a real fire scenario. The results are shown in Figure 4 and detailed data are given in Table 2. The total heat release (THR) and the peak of heat release rate (PHRR) of pure WPU are 84.20 MJ/m^2^ and 452.5 kW/m^2^, respectively, indicating that pure WPU released a large amount of heat and made it easy to cause the “flashover” phenomenon. The BP/BN/WPU exhibits the best flame-retardant properties, with a 50.94% decrease in PHRR (Figure 4a) and 23.92% decrease in THR (Figure 4b), respectively. The introduction of BP and BN nanosheets into WPU significantly reduced heat release and restricted the “flashover” phenomenon. The time to PHRR (TPHRR) and time to ignition (TTI) values of the materials are also shown in Table 2. The TPHRR of BP/BN/WPU arrived last of all. That is to say the burning time of the material was increased, which is a benefit for escaping, rescuing, and firefighting. The TTI of pure WPU and BN/WPU have bigger values than pure BN/WPU and BP/BN/WPU, which indicates that the addition of BP makes the polymer matrix easy to ignite. This phenomenon is a common feature of phosphorus-based flame retardants [33,34]. The reason may be that the addition of phosphorus results in the composite polymers having a lower decomposition temperature, leading them to release inflammable gases and ignite earlier. The average of the effective heat of combustion (av-EHC) reveals the volatiles dilution in the gas phase, and usually also discloses the inhibition of gas phase combustion by flame retardants. As shown in Table 2, the av-EHC of WPU and BN/WPU are not much different. This indicates that BN mainly plays a role in the condensed phase rather than the gas phase. Compared with WPU, the av-EHC of BP/WPU and BP/BN/WPU is much lower. The low av-EHC is due to the capture of gas phase radicals by black phosphorus during combustion.

The CO_2_ release (Figure 4c) and the CO release (Figure 4d) curves indicate that CO_2_ is the main released gas, with little difference in release amount during the burning process. However, the CO release amount of BP/WPU is the highest, and the CO/CO_2_ ratio was increased due to added BP, which indicates that BP works in the gas phase with the ability to restrict the complete combustion of polymer.

The flame retardant effectivity (EFF) and synergistic effectivity (SE) are used to numerically evaluate the synergistic effect of multi-component flame retardant systems [35,36]. Flame retardant effectivity (EFF) and synergistic effectivity (SE) were calculated from CC data as follows: EFF = (PHRR_polymer_ − PHRR_composite_)/Flame-retardant content; SE = EFF_Flame-retardant_ + _synergists_/EFF_Flame-retardant_. BP/BN/WPU has the highest EEF value, indicating that compared with the single flame retardant, BP and BN synergistic flame retardant has the highest flame retardant efficiency. The SE values of BP/WPU and BN/WPU materials are 1.15 and 1.5, respectively, indicating that BP and BN can synergistically improve the flame retardancy of WPU.

### 3.4. Flame-Retardant Mechanism of BP/BN/WPU

#### 3.4.1. Products Analysis of Carbon Residue

**Figure 5:**
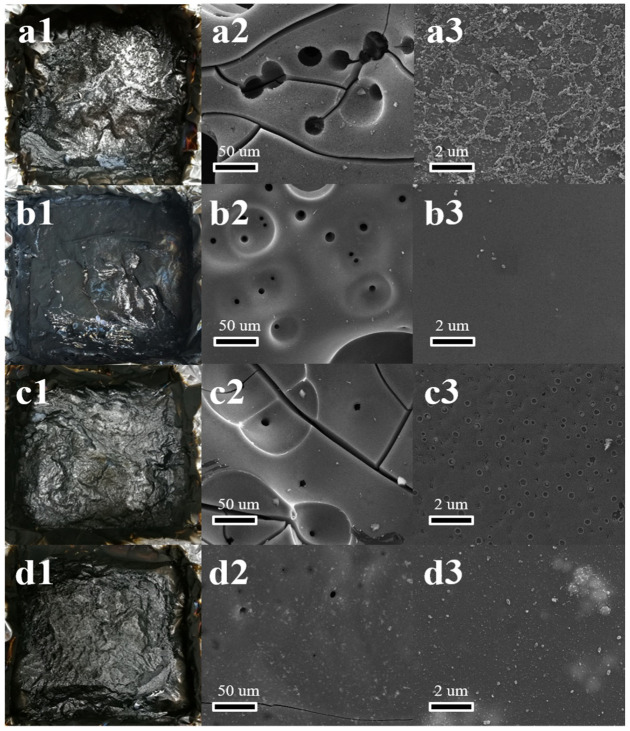
The images of the residues after the CC test: (**a1**,**b1**,**c1,d1**) the real picture of the pure WPU, BP/WPU, BN/WPU, BP/BN/WPU, respectively; (**a2**,**b2**,**c2**,**d2**) SEM image of the pure WPU, BP/WPU, BN/WPU, BP/BN/WPU, respectively; (**a3**,**b3**,**c3**,**d3**) the enlarged SEM image of the pure WPU, BP/WPU, BN/WPU, BP/BN/WPU.

The residues after the CC tests were used to analyze the products after combustion, and the corresponding results are summarized in Table 3. The SEM images were used to observe the microstructure of the residues (Figure 5). The pure WPU left scarcely any residue (Table 3). Both the addition of BP and BN could enhance the residues of WPU, and the BP/BN/WPU further increase the residues up to 10.34%. This result was also confirmed by the digital images of residues in Figure 5a1–d1. Figure 5a2 shows the residue of the pure WPU presents a lot of large cracks and ~10 µm holes. The enlarged image (Figure 5a3) shows that the residue of WPU is shaggy in shape with discontinuous particles. This structure usually facilitates heat transformation and the release of inflammable gas. After the addition of BP and BN, the BP/WPU and BN/WPU show obvious structure difference with relatively smaller holes and smoother surface (Figure 5b3,c3). The residue of BP/BN/WPU exhibited a completely dense surface without small holes, which indicates the good synergistic effect between the BP and BN nanosheets in triggering the catalytic carbonization and constructing a barrier.

EDS analysis was carried out to confirm the different roles of BP and BN in the flame retardant. As shown in Table 3, the element P content in the residue of BP/WPU was only 0.0086 g, indicating that 90.25% of BP is consumed during combustion. The element B in the residue of BP/BN/WPU was 0.060 g. which means most BN was retained and functioned in the condensed phase. According to a previous study on the flame retardant properties of phosphorus [37], BP may also play a key role in the gas phase because it can be converted into P–O radicals and diffuse in the surrounding gas, which can then react with the H or OH radicals generated by polymers under the burning conditions, consequently reducing the energy of the flame. BN is stable and will accumulate in the condensed phase, which can form a physical barrier to reduce heat transfer and release of combustible gases [38,39,40].

**Figure 6:**
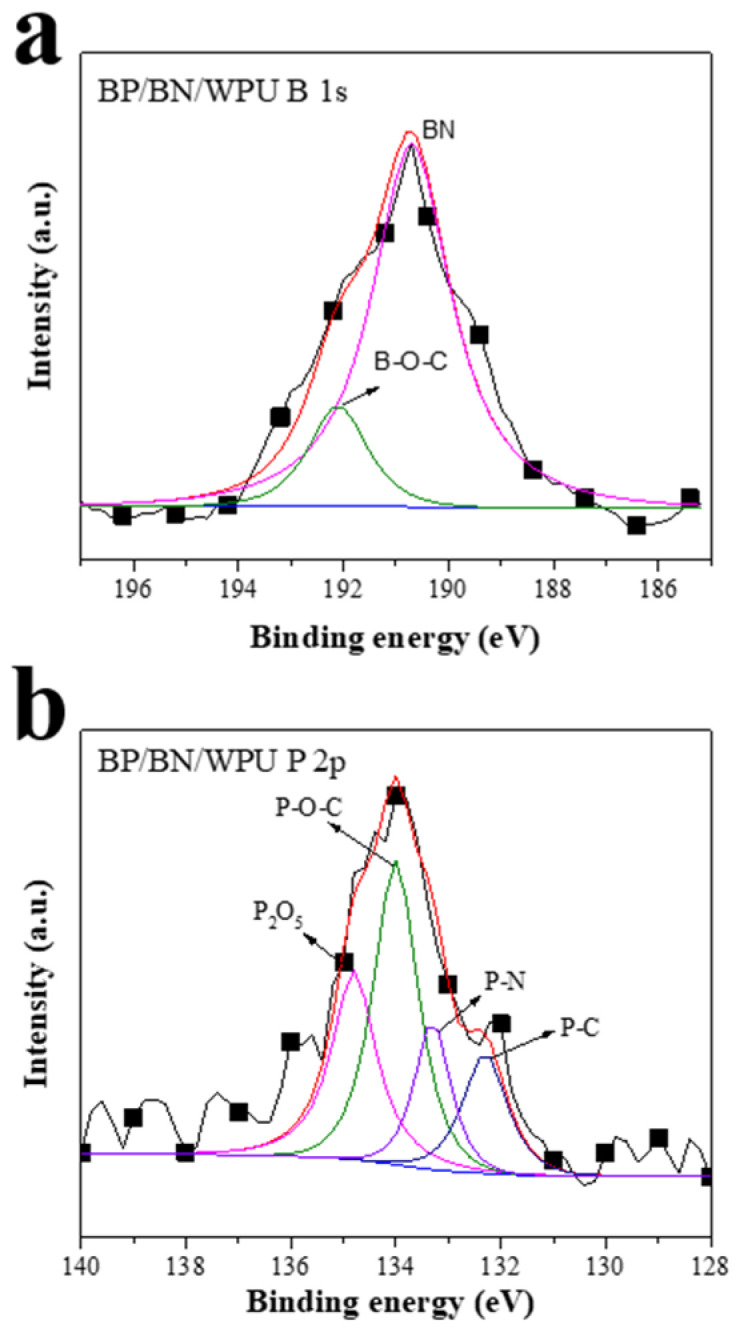
XPS spectra of the residue of BP/BN/WPU composite material after CC test; (**a**) high-resolution XPS spectra of B 1s of the residue of BP/BN/WPU composite material; (**b**) high-resolution XPS spectra of P 2p of the residue of BP/BN/WPU composite material.

In order to further analyze the component of BP in the residue after the CC test, XPS was conducted on the residue powder of BP/BN/WPU. The high-resolution P1s XPS of BP/BN/WPU (Figure 6a) can be deconvoluted into three peaks with binding energies at 132.3, 134.0 and 134.8 eV, corresponding to P–C, P–O–C and phosphoric anhydride (P_2_O_5_) [41], respectively. Figure 6b shows the high-resolution XPS spectra of B 1s, which was deconvoluted into two peaks with binding energies at 190.7 and 192.2 eV corresponding to the B–N, B–O–C. Boron element mainly exists in BN form in the residue, and a small amount of B forms chemical bonds with C. This result indicates that BP can be converted into phosphoric acid and phosphoric anhydride at high temperature, which can promote the polymers to produce a carbon layer. In addition, BN also play a catalytic role in carbon formation. The generated carbon layer and the amount of BN left behind will form an insulating layer to prevent the materials from contacting with oxygen and heat transferring, consequently weakening the fire [42,43].

#### 3.4.2. The Product Analysis of the Gas Phase

**Figure 7:**
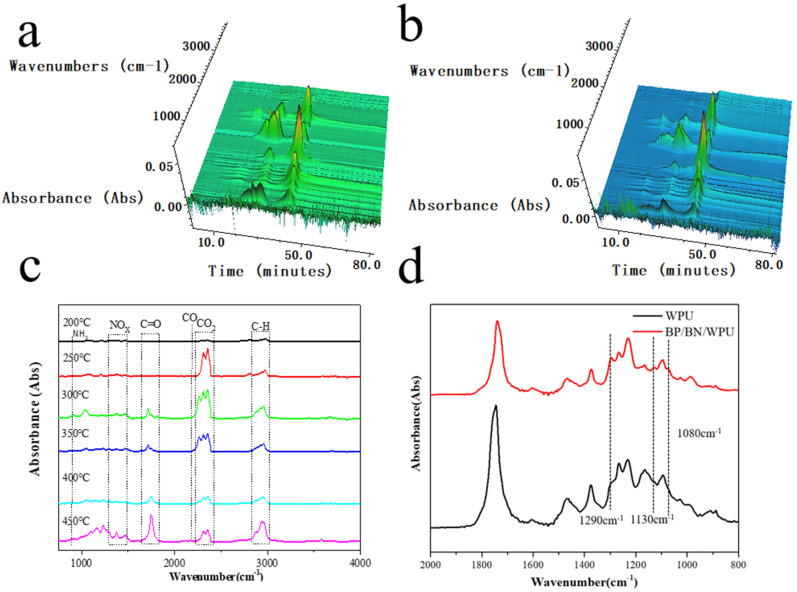
(**a**) 3D image of FTIR spectra of the WPU; (**b**) 3D image of FTIR spectra of the BP/BN/WPU; (**c**) The FTIR spectra of BP/BN/WPU obtained at different temperatures; (**d**) The enlarged image for comparison of the biggest mass loss in the second TG stages of the pure WPU and BP/BN/WPU.

In order to determine the pyrolytic mechanism, the decomposition process of BP/BN/WPU was analyzed by Thermogravimetric analysis–Fourier transform infrared spectroscopy (TG–FTIR). The 3D map of the FTIR spectra shows BP/BN/WPU releases CO_2_ earlier than pure WPU (Figure 7a,b). The results indicated that the addition of BP/BN can release non-combustible gas in the initial thermal decomposition of WPU, which can dilute the oxygen from the air and dilute combustible gases from the material. 

To further study the thermal degradation process, the chemical structure changes of the BP/BN/WPU at different pyrolysis temperatures were investigated (Figure 7c). The changes in characteristic peaks reflect the detailed decomposition process of WPU and WPU composites. The peak at 2800–3100 cm^−1^ could be attributed to the stretching vibration of C–H [44,45]. The peaks at 2250–2400 cm^−1^, 2190 cm^−1^ and 1650–1810 cm^−1^ are related to CO_2_, CO, and carbonyl compound, respectively. The peak at 915 cm^−1^ corresponds to the bending vibration of the NH_3_, and peaks at 1320–1550 cm^−1^ refer to the stretching vibration of NO_x_ (such as N_2_O, NO and NO_2_) [46], indicating WPU combustion has two main stages: Before the combustion, the WPU decomposed to give flammable gases such as hydrocarbon and carbonyl compounds. With the increase of temperature, a large amount of combustible gas was ignited and the WPU began to burn violently. The FT-IR spectra of the BP/BN/WPU sample (Figure 7d) show a significant enhancement at 1290, 1130 and 1080 cm^−1^ compared to WPU, which could be explained by the P=O, PO_2_– and P–O groups [47,48]. The presence of phosphorus-containing gas should be attributed to the formation of phosphorus-containing radicals from the decomposition of BP, which demonstrates the significant role of BP in the gas-phase flame retardancy.

#### 3.4.3. Synergistic Flame-Retardant Mechanisms

**Figure 8:**
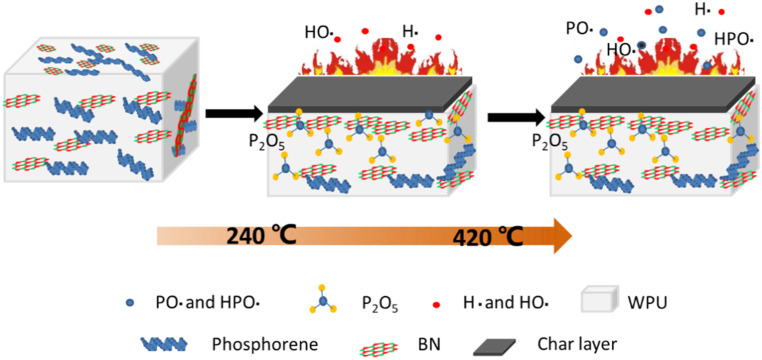
Schematic illustration of the flame-retardant mechanism.

Based on the above flame-retardant performances and analyses, a possible flame retardant mechanism is proposed in Figure 8. At high temperature or in a real fire scenario, BP starts to decompose at about 240 °C. When the temperature reaches 420 °C or higher, most of the BP begins to enter the gas phase. The BP nanosheets work in both of the gaseous and condense phases for fire restriction. On the one hand, most of the BP will form radicals in the gas phase by absorbing surrounding oxygen and hydrogen atoms, which react with the pyrolytic radicals of the matrix polymers to inhibit the chain reaction. At the same time, the liberated incombustible gases will dilute the combustible gases and reduce their contact with oxygen. On the other hand, the residual BP will capture the oxygen in the polymers and transform it into phosphoric anhydride, which will promote the formation of a char layer and prevent the release of carbon-containing gas. BN nanosheets mainly remain in the condensed phase, synergistically catalyze carbon, and build a nano-barrier formation with black phosphorus due to the fact that they have good thermo-stability and a peculiar spatial three-dimensional network structure. Therefore, the high efficiency flame retardant performance of BP/BN can be attributed to the synergy effects of the gas phase mechanism of black phosphorus and the condensation phase mechanism of boron nitride during the combustion process.

## 4. Conclusions

BP and BN nanosheets were used as a flame retardant for WPU. The SEM and mapping results indicated that the BP and BN nanosheets were distributed uniformly in the matrix WPU. The flame-retardant tests demonstrated that the PHRR of WPU decreases by 50.94% and the THR decreases by 23.92% at a BP/BN content of only 0.4 wt%. The LOI of the BP/BN/WPU composite increased from 21.7% to 33.8%, compared with pure WPU. The residue of BP/BN/WPU after the CC test was denser and approximately 10 times more than the residue of pure WPU, according to TG, SEM, XPS, TG-IR analysis of WPU during the combustion process. Efficient flame retardancy is due to the synergistic effects of BP and BN. To conclude, BP/BN nanosheets can provide a good flame retardant effect with very low addition amount, and have a good application prospect in the field of flame retardancy.

## Figures and Tables

**Table 1:** The additive amount of the BP and BN nanosheets in the samples.

Samples	Weight (g)	Percentage of BP (%)	Percentage of BN (%)	Content of BP (g)	Content of BN (g)
WPU	22.1	0	0	0	0
0.4BP/WPU	22	0.40	0	0.0880	0
0.4BN/WPU	22.4	0	0.40	0	0.0896
0.2/0.2BP/BN/WPU	22.2	0.20	0.20	0.0444	0.0444

**Table 2:** The cone calorimeter test data of WPU, G/WPU, and BP/G/WPU.

Sample	TTI (s)	TPHRR (s)	PHRR (kW/m^2^)	THR (MJ/m^2^)	Av-EHC (MJ/kg)	CO/CO_2_	EFF	SE
WPU	65	297	452.5	84.20	31.07	0.021		
BP/WPU	40	257	252.7	62.57	29.29	0.056	499.5	1.15
BN/WPU	62	264	308.3	72.25	31.30	0.027	360.5	1.5
BP/BN/WPU	51	305	222.0	64.06	27.9	0.037	576.25	

**Table 3:** The content of BP and BN nanosheets in the residues after the CC test.

Residue Samples	Weight (g)	Total Residues (%)	Condensed Phase of P (%)	Condensed Phase of BN (%)	Gas Phase of P (%)	Gas Phase of BN (%)
WPU	0.20	0.1	0	0	0	0
0.4%BP/WPU	1.3	6.34	9.75	0	90.25	0
0.4%BN/WPU	2.1	7.38	0	66.96	0	33.04
0.2%/0.2%BP/BN/WPU	2.4	10.34	19.46	68.69	80.54	31.31
